# Hydrostatic Pressure and Temperature Measurements Using an In-Line Mach-Zehnder Interferometer Based on a Two-Mode Highly Birefringent Microstructured Fiber

**DOI:** 10.3390/s17071648

**Published:** 2017-07-18

**Authors:** Gabriela Statkiewicz-Barabach, Jacek Olszewski, Pawel Mergo, Waclaw. Urbanczyk

**Affiliations:** 1 Department of Optics and Photonics, Faculty of Fundamental Problems of Technology, Wroclaw University of Science and Technology, 50-370 Wroclaw, Poland; jacek.olszewski@pwr.edu.pl (J.O.); waclaw.urbanczyk@pwr.edu.pl (W.U.); 2 Laboratory of Optical Fiber Technology, Maria Curie-Sklodowska University, 20-031 Lublin, Poland; pawel.mergo@poczta.umcs.lublin.pl

**Keywords:** intermodal interferometer, microstructured fiber, hydrostatic pressure, temperature measurement

## Abstract

We present a comprehensive study of an in-line Mach-Zehnder intermodal interferometer fabricated in a boron-doped two-mode highly birefringent microstructured fiber. We observed different interference signals at the output of the interferometer, related to the intermodal interference of the fundamental and the first order modes of the orthogonal polarizations and a beating of the polarimetric signal related to the difference in the group modal birefringence between the fundamental and the first order modes, respectively. The proposed interferometer was tested for measurements of hydrostatic pressure and temperature for different alignments of the input polarizer with no analyzer at the output. The sensitivities to hydrostatic pressure of the intermodal interference signals for *x*- and *y*-polarizations had an opposite sign and were equal to 0.229 nm/MPa and −0.179 nm/MPa, respectively, while the temperature sensitivities for both polarizations were similar and equal 0.020 nm/°C and 0.019 nm/°C. In the case of pressure, for the simultaneous excitation of both polarization modes, we observed a displacement of intermodal fringes with a sensitivity depending on the azimuth of the input polarization state, as well as on the displacement of their envelope with a sensitivity of 2.14 nm/MPa, accompanied by a change in the fringes visibility. Such properties of the proposed interferometer allow for convenient adjustments to the pressure sensitivity of the intermodal fringes and possible applications for the simultaneous interrogation of temperature and pressure.

## 1. Introduction

In recent years, traditional fiber-optic Mach-Zehnder interferometers (MZIs) with two separated reference and sensing arms have been replaced in many applications by simpler in-line fiber interferometers. Fiber-optic couplers, necessary to split and recombine a guided light, have been replaced in the in-line MZIs by coupling points splitting or recombining light guided in the fundamental core mode and the cladding, or higher order modes. One can distinguish between two different arrangements of this type of in-line interferometer, which are made on a single piece of fiber or spliced from several pieces of fibers of different modes. One of the possible designs of the MZI in one piece of fiber is based on a pair of long period gratings (LPGs) acting as modal couplers [1,2,3,4]. However, in such cases, both LPGs should be identical to obtain the maximum performance of the MZI [3]. Fiber tapering, or collapsing air holes in the microstructured fiber is, compared to LPGs, a relatively simple and fast method of making coupling points [5,6,7,8]. Additionally, in such an approach, the interference fringes are not limited to the spectral range of the LPGs. Some drawbacks to these methods are weaker fiber strength at the tapered regions and high insertion loss at the collapsing points [7]. Another cost-effective and fast method of making coupling points is to splice fibers of different core sizes, for example, a fiber with a smaller mode field diameter is inserted between two conventional single mode fibers (SMFs) [9]. One can also splice a piece of a fiber between two different (or the same fibers) with lateral offset [7,10,11]. In this case, the number of excited cladding modes can be controlled by adjusting the fibers’ lateral shift. However, to obtain a high excitation coefficient of the cladding modes, the core-offset has to be significant, which results in relatively high insertion loss [11]. In the literature, one can also find studies of the formation of in-line MZIs by combining two of the abovementioned methods, for example, devices based on a taper and an LPG [12], a core-offset and an LPG [13], or a core-offset and a taper [14].

Due to flexible configurations, small size, and high sensitivity and resolution, a possible range of applications of in-line intermodal Mach-Zehnder interferometers cover various types of devices for measuring different physical parameters, such as temperature [6,8,9,14], strain [8,14], or refractive index change [5,6]. Simultaneous measurements of temperature and strain were reported by using an in-line MZI with a pair of LPGs made in double cladding fiber [4], an interferometer fabricated in a boron-doped highly birefringent microstructured fiber with two tapered regions [8], and an MZI with a core-offset and taper in a single mode fiber [14]. Moreover, simultaneous measurements of temperature and refractive index using the MZI made in a single mode fiber were reported in Reference [6].

In this paper, we present for the first time a comprehensive study of the sensitivity to hydrostatic pressure and temperature of the in-line Mach-Zehnder fiber interferometer fabricated in a boron-doped two-mode highly birefringent microstructured optical fiber (HB MOF) using a fusion splicer. The characterized sensor was comprised of an HB MOF segment spliced with two single mode leading fibers and a linear polarizer placed at the sensor input. A small lateral shift of the HB MOF and the leading-in single mode fiber allowed for the excitation of the fundamental and the first order mode guided in the HB MOF, and resulted in the intermodal interference observed in the spectral domain at the sensor output. Spectral interferograms were acquired for the different azimuths of the input polarization state with no polarizer at the sensor output and analyzed for different values of applied temperature and hydrostatic pressure. The proposed sensor showed several unusual features such as the opposite sign of sensitivity to hydrostatic pressure for *x*- and *y*-polarized intermodal interference signals and the possibility of tuning the intermodal sensitivity to hydrostatic pressure by adjusting the input polarization state. Such properties of the proposed sensor allowed for simultaneous measurements of temperature and hydrostatic pressure, or other parameters by interrogating the visibility and displacement of the intermodal interference fringes and their envelope.

## 2. The Principle of the Sensor Operation

**Figure 1:**
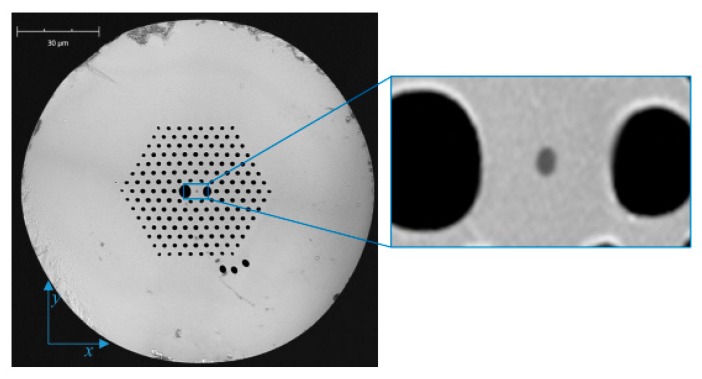
Scanning Electron Microscope (SEM) image of the birefringent microstructured optical fiber with boron-doped inclusion in the center of the core (darker spot) used for the fabrication of the in-line Mach-Zehnder interferometer.

For the fabrication of the proposed in-line Mach-Zehnder interferometer, we used a boron-doped birefringent microstructured fiber as shown in Figure 1, which was fabricated in the Laboratory of Optical Fiber Technology, University of Marie-Curie Sklodowska (UMCS) in Lublin, Poland. The birefringence in this fiber was induced by two large holes located symmetrically with respect to the core. The fiber geometrical parameters averaged for the first two layers of holes surrounding the core were as follows: pitch distance 3.9 μm; diameter of cladding holes 1.75 μm; size of large holes 4.5 μm × 4.0 μm and 3.6 μm × 3.0 μm; external diameter of the fiber 127 μm; and the size of the boron-doped inclusion was 0.68 μm × 0.92 μm. The boron concentration in the inclusion was equal to 13 mol %. It is worth mentioning that a similar construction fiber has been used previously to fabricate an intermodal interferometer for simultaneous strain and temperature measurements [8].

Deeper insight into the operation principle of the intermodal interferometer requires knowledge of the modal properties of the boron-doped fiber, which was used for the sensor fabrication. We numerically simulated the fiber properties by means of the finite-element method (FEM) implemented in a commercially available COMSOL Multiphysics software [15]. In our model, the mesh was generated using the geometry obtained by the digital processing of an SEM image of the investigated fiber cross-section (Figure 1). It was assumed that the refractive indices of pure silica glass and boron-doped glass satisfied the dispersion relations given in References [16,17]. By solving the fully-vector wave equation for the defined fiber structure, we were able to determine the complex effective refractive indices for fundamental LP_01_ and the first high-order LP_11_ polarization modes as a function of wavelength. In the next step, we calculated the spectral dependences of the group modal birefringence and the difference between the group effective indices of the first order and the fundamental modes. We found that the simulation results depended on the procedure used to process an image of the fiber cross-section, namely the selection of a binarization threshold which influenced the diameter of air-holes and the boron-doped inclusion in the fiber model. In the SEM image, the edges of the air-holes and inclusion were slightly blurred; therefore, it was difficult to correctly set the threshold value. We adjusted the threshold by matching the results of the simulations to the measured values.

**Figure 2:**
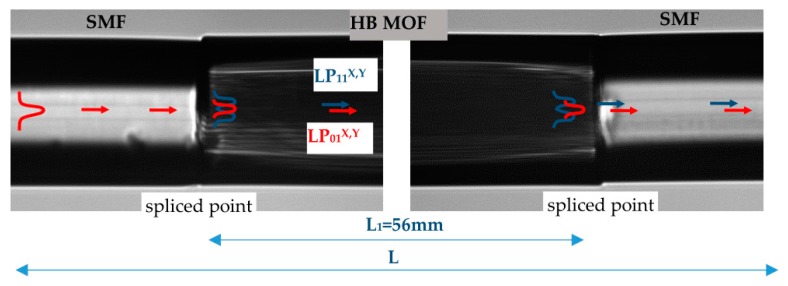
Microscope image and schematic modes distribution in the proposed in-line interferometric sensor composed of 56 mm of HB MOF spliced with leading-in SMF-28 fibers.

The operation principle of the proposed interferometer is schematically illustrated in Figure 2, and was composed of a 56-mm long HB MOF spliced with a lateral shift with two pieces of Corning SMF-28 fiber. The splices were made using an Ericsson fusion splicer. We controlled the contrast of the intermodal interference when aligning the fibers’ lateral shift before making each splice. The maximum contrast in the spectral range of 1400–1700 nm was obtained for the lateral offset of about 2 μm. Due to the lateral shift and a slight collapse of the microstructured region at both splices, an insertion loss slightly higher than 3 dB was observed at each splice. At the first splice, the fundamental LP_01_ mode was coupled to the LP_11_ higher order mode, and at the second splice both modes were recombined producing the interference signal, which depended on the polarizer alignment at the input of the leading-in fiber.

If the linear polarization state (controlled by the polarizer at the input of the leading-in fiber) is aligned in parallel to one of the symmetry axes of the highly birefringent fiber, we excite in the HB MOF only one pair of polarization modes and as a result, the interference signal produced by the pair of modes $LP_{01}^{x}$ and $LP_{11}^{x}$ or $LP_{01}^{y}$ and $LP_{11}^{y}$ can be expressed by:

(1) $$I_{x}\left(\lambda \right)=I_{0}\cos^{2}\alpha \left(1+\gamma \cos\frac{2\pi (n_{01}^{x}-n_{11}^{x})L}{\lambda }\right)$$

and

(2) $$I_{y}\left(\lambda \right)=I_{0}\sin^{2}\alpha \left(1+\gamma \cos\frac{2\pi (n_{01}^{y}-n_{11}^{y})L}{\lambda }\right)$$

where α is the azimuth of the input polarization state and γ is the fringe contrast depending on the fibers’ lateral offset at splices; finally, L is the length of the HB MOF.

**Figure 3:**
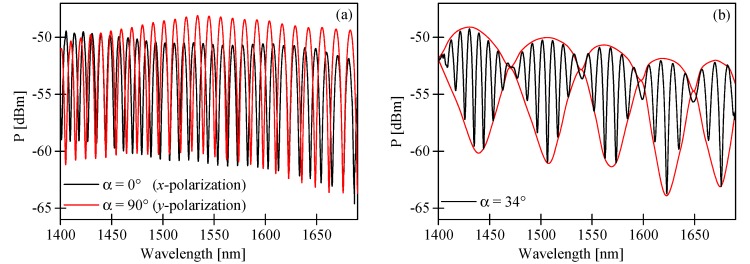
Spectral interference fringes at the output of an in-line intermodal interferometer based on a 56-mm long two-mode HB MOF for excited *x*-polarization (black line) and *y*-polarization (red line) (**a**) and both polarization modes by setting the azimuth of the input polarization state at α = 34° (**b**).

The periodicity of the interference fringes observed in the output spectrogram depends on the difference in the group refractive indices of the fundamental and the first order modes for *x*- and *y*-polarization, respectively (Figure 3a). The phase shift ∆ϕ(λ) between the fundamental and the first order modes can be expressed as:

(3) $$\Delta \varphi \left(\lambda \right)=\frac{2\pi (n_{01}^{x,y}-n_{11}^{x,y})L}{\lambda }$$

After the differentiation of the above equation and performing rather straightforward calculations, one can easily obtain an expression for the group index difference of the fundamental and first order mode, respectively, for *x*- or *y*-polarization:

(4) $$N_{01}^{x,y}-N_{11}^{x,y}=-\frac{\lambda^{2}}{2\pi L}\frac{d\Delta \varphi }{d\lambda }$$

**Figure 4:**
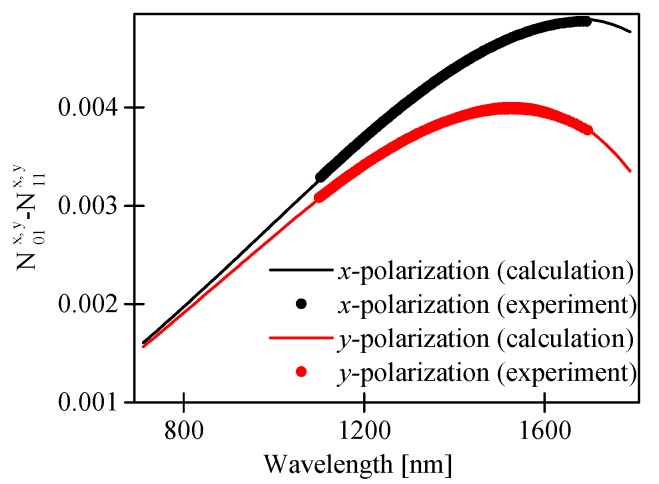
Calculated and measured difference in group effective indices of the LP_01_ and LP_11_ modes for both polarizations.

In Figure 4, we compared the calculated and the measured difference in the group refractive indices of the fundamental and the first order modes for both polarizations, i.e., $N_{01}^{x,y}-N_{11}^{x,y}$. The measured and the calculated values of $N_{01}^{x,y}-N_{11}^{x,y}$ showed very good agreement, which confirms the validity of our numerical model.

If the azimuth of the input polarization state is set at a certain angle α to the polarization axes of the HB MOF, both polarization modes are excited in this fiber. As a result, we observed the superposition of two interference signals produced by two pairs of the orthogonally polarized modes at the output of the leading out fiber, which can be represented by the following formula:

(5) $$I\left(\lambda \right)=I_{0}\left[1+\gamma \cos^{2}\alpha \cos\left(\frac{2\pi (n_{01}^{x}-n_{11}^{x})L}{\lambda }\right)+\gamma \sin^{2}\alpha \cos\left(\frac{2\pi (n_{01}^{y}-n_{11}^{y})L}{\lambda }\right)\right]$$

After performing rather straightforward derivations, one can write:

(6) $$I\left(\lambda \right)=I_{0}\left[1+\gamma \cos\left(\frac{2\pi (n_{01}^{a}-n_{11}^{a})L}{\lambda }\right)\cos\left(\frac{\pi (B_{01}^{}-B_{11}^{})L}{\lambda }\right)-\gamma \cos2\alpha \sin\left(\frac{2\pi (n_{01}^{a}-n_{11}^{a})L}{\lambda }\right)\sin\left(\frac{\pi (B_{01}^{}-B_{11}^{})L}{\lambda }\right)\right]$$

where $n_{01}^{a}$ and $n_{11}^{a}$ are the effective refractive indices of the fundamental and the first order modes averaged over polarization:

(7) $$n_{01}^{a}=\frac{n_{01}^{x}+n_{01}^{y}}{2},\;n_{11}^{a}=\frac{n_{11}^{x}+n_{11}^{y}}{2}$$

and B_01_ and B_11_ are the phase modal birefringences of the fundamental and the first order mode:

(8) $$B_{01}=n_{01}^{x}-n_{01}^{y},\;B_{11}=n_{11}^{x}-n_{11}^{y}$$

If the azimuth of the input polarization state is aligned at α = 45° with respect to the fiber polarization axis, Equation (6) takes the following form:

(9) $$I\left(\lambda \right)=I_{0}\left[1+\gamma \cos\left(\frac{2\pi (n_{01}^{a}-n_{11}^{a})L}{\lambda }\right)\cos\left(\frac{\pi (B_{01}-B_{11})L}{\lambda }\right)\right]\text{​}$$

**Figure 5:**
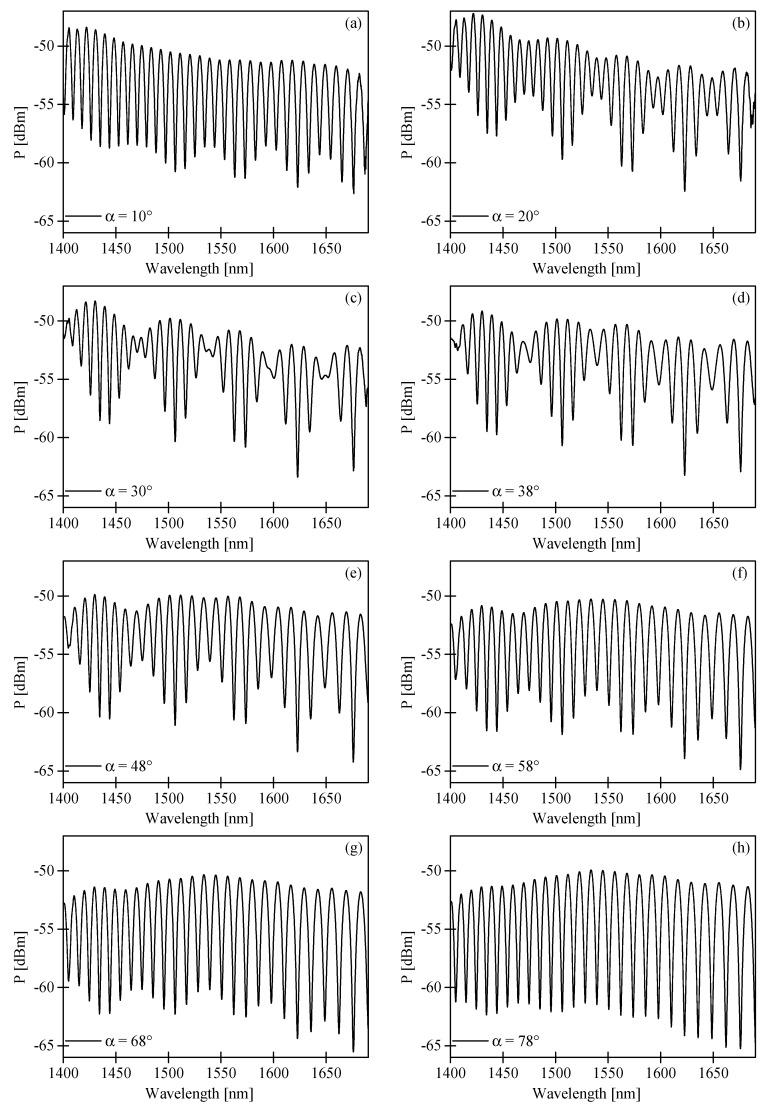
Spectral interference fringes at the output of the in-line intermodal interferometer based on the 56-mm long HB MOF registered for different azimuth of the input polarization state: α = 10° (**a**); α = 20° (**b**); α = 30° (**c**); α = 38° (**d**); α = 48° (**e**); α = 58° (**f**); α = 68° (**g**); α = 78° (**h**).

The first cosine term in the above formula represents the intermodal interference fringes averaged over polarization, while the second term represents the envelope determined by the differences in birefringence of the fundamental and the first order modes. The corresponding terms are indicated by black and red lines in Figure 3b, which show the output spectrogram produced by the 56-mm long HB MOF. As per Equation (6), the visibility of the envelope depends on the azimuth of the input polarization and reaches the highest value at α = 45°. In Figure 5, we present the experimental spectrograms registered at the sensor output for different α. One should note that the alignment of the input polarization state assuring the maximum visibility of the envelope is different from the 45° predicted in Equation (6). In Figure 5e, we display the spectrogram registered for α = 48°, which does not differ much from the spectrogram obtained for α = 45°, showing a relatively low contrast of the envelope. Clearly visible in Figure 5c, setting the input polarization state azimuth at α = 30° yielded the maximum modulation depth of the envelope in the spectral range 1600–1650 nm. The discrepancy between the theoretical model and the experimental observations is not fully understood, and may be caused by several factors, including: the unequal excitation of *x*- and *y*-polarized LP_01_ and LP_11_ modes at the laterally shifted splices, the polarization-dependent loss (PDL) of the used HB MOF [18], or the spectral dependence of the coupling coefficients at the splices.

We also compared the difference in group modal birefringence between the fundamental and the first order modes G_01_–G_11_ calculated by the FEM method and determined from the spectrogram shown in Figure 3b. The positions of the minima and maxima of the interference pattern envelope were approximated by the red curve shown in Figure 3b. In this case, the phase shift ∆ϕ(λ) in the registered spectrogram can be expressed as:

(10) $$\Delta \varphi \left(\lambda \right)=\frac{2\pi (B_{01}-B_{11})L}{\lambda }$$

For the experimental evaluation of the group birefringence difference, we used the following expression:

(11) $$G_{01}-G_{11}=-\frac{\lambda^{2}}{2\pi L}\frac{d\Delta \varphi }{d\lambda }.$$

**Figure 6:**
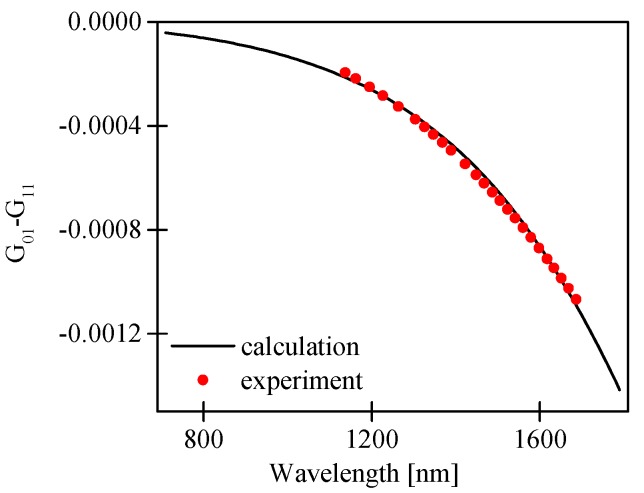
Comparison of measured and calculated difference in the group modal birefringence G_01_–G_11_ of the LP_01_ and LP_11_ modes in the investigated HB MOF with boron-doped inclusion.

The measured and calculated values of G_01_–G_11_ (presented in Figure 6) showed very good agreement. 

## 3. Sensing Characteristics

To measure the sensitivity to temperature, the proposed interferometer was placed in a water heating system equipped with a thermocouple and exposed to increasing and decreasing temperature cycles in a range of 20–100 °C. The ambient temperature was measured by the thermocouple with the accuracy of 0.1 °C. To determine pressure sensitivity, the interferometer was installed in a specially designed pressure chamber filled with oil and subjected to several pressure cycles in a range from 0.1 (atmospheric pressure) to 10 MPa with an accuracy of 0.1 MPa. To avoid the possible influence of bending on the sensitivity measurements, the fiber was kept straight during all experiments. Depending on the azimuth of the polarization state at the input of the interferometer, we observed a variation of the interference fringe visibility, the fringe displacement, and the envelope displacement in response to changes in hydrostatic pressure and in temperature applied to the interferometer. The displacement of the interference fringes was caused by changes in the effective refractive indices of the fundamental and the first order mode for both polarizations $n_{01}^{x,y}$ and $n_{11}^{x,y}$and in the interferometer length L [8], while the shift of the envelope was related to the variation of the birefringence difference of the fundamental and the first order mode. As the displacement of the intermodal fringes is scaled proportionally with the interferometer length L while the spectral separation of the fringes is inversely proportional to L, care has to be taken when selecting the interferometer length L, which would assure unambiguous fringe tracking in the full measurement range of temperature and pressure. For this reason, the interferometer length L was set at 56 mm in our experiments.

**Figure 7:**
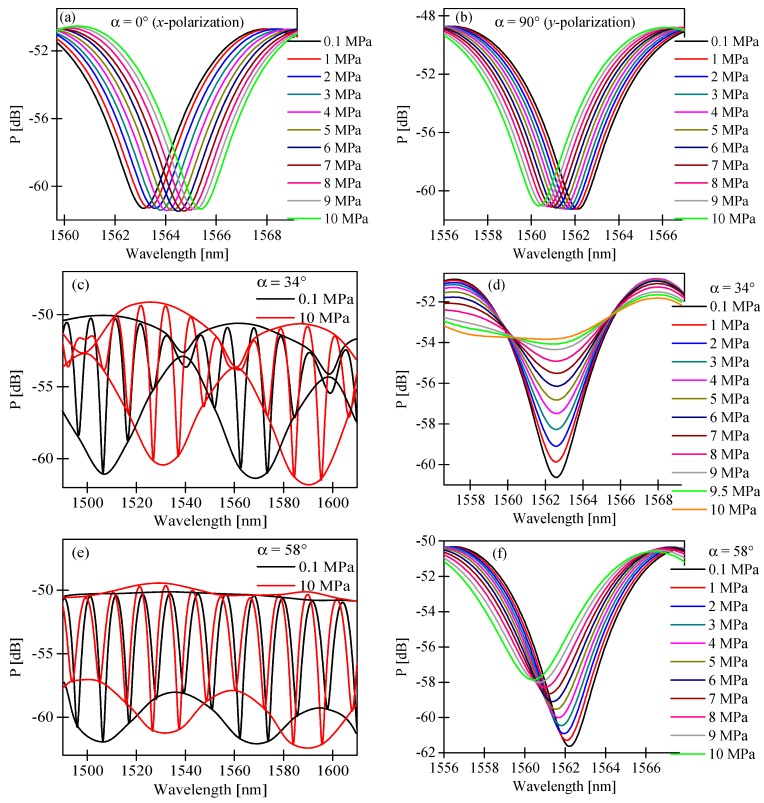
Evolution of the intermodal interference fringe with the intensity minimum close to 1563 nm registered for increasing hydrostatic pressure in the range of 0.1–10 MPa for the azimuth of the input polarization state set at α = 0° (**a**); α = 90° (**b**); α = 34° (**d**); and α = 58° (**f**); and output spectrograms registered in the range from 1490 nm to 1610 nm for selected values of hydrostatic pressure showing the displacement of the envelope for the azimuth of the input polarization state set at α = 34° (**c**); and α = 58° (**e**).

**Figure 8:**
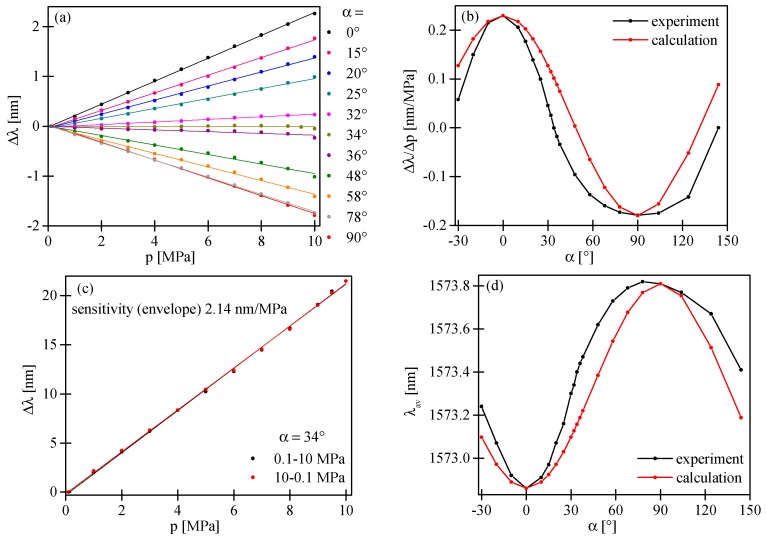
Displacement of the intermodal interference fringe with an intensity minimum close to 1563 nm versus the applied pressure for different azimuths of the input polarization state (**a**) and a comparison of the experimental and theoretical value of the sensitivity to pressure upon the azimuth of the input polarization state (**b**). Displacement of the envelope versus applied pressure (**c**) and experimental and theoretical dependence of the position of the interference minimum at zero pressure and the intermodal sensitivity to pressure upon the azimuth of the input polarization state (**d**).

If the input polarization state is in parallel alignment to one of the symmetry axes of the HB MOF, we observed in the output spectrogram the interference fringes with periodicity depending on the difference in the group refractive indices of the fundamental and the first order mode for *x*- or *y*-polarization. As a consequence, the variation of the measurand applied to the HB MOF resulted only in a shift of the intermodal fringes. As shown in Figure 7a,b, the intermodal fringes moved towards shorter wavelengths for *x*-polarized light and towards longer wavelengths for *y*-polarized light in response to increasing pressure. The displacement of the fringe with an intensity minimum close to 1563 nm was linear in the investigated pressure range with no trace of hysteresis. The sensitivities to pressure had an opposite sign and were equal to $K_{p}^{x}$ = 0.229 nm/MPa and $K_{p}^{y}$ = −0.179 nm/MPa for *x*- and *y*-polarization, respectively (Figure 8a; black and red points). 

If the input polarization state is aligned at a certain angle α to the symmetry axes of the HB MOF, we excited both polarization modes at the input of the interferometer. As a consequence, we observed (as no analyzer was used) at the sensor output the superposition of two intermodal interference signals produced by two pairs of the orthogonally polarized LP_01_ and LP_11_ modes. We performed sensitivity measurements of the intermodal interference fringes for different azimuths of the input polarization state α (Figure 8a). For this purpose, we monitored the pressure-induced displacement of the intermodal interference fringes of the highest contrast. As shown in Figure 8b, the value of the intermodal sensitivity was strongly dependent on the azimuth of the input polarization state, while the sensitivity of the envelope remained unchanged and equaled 2.14 nm/MPa (Figure 8c). It is worth mentioning that to determine the envelope shift, we used the procedure presented in Reference [19]. The dependence of the intermodal sensitivity upon the azimuth of the input polarization state can be explained using the approach outlined below. In the vicinity of the interference minimum, a parabolic approximation of intensity can be used to describe the fringe position versus applied pressure:

(12) $$I^{x,y}\left(\lambda ,p\right)=I_{0}{\left(\lambda -\lambda_{0}^{x,y}-K_{p}^{x,y}p\right)}^{2}\gamma +\text{const}$$

for the interference of *x*- and *y*-polarized LP_01_ and LP_11_ modes, respectively. In the above equation, $\lambda_{0}^{x,y}$ indicates the initial position of the intensity minimum at zero pressure, $K_{p}^{x,y}$ is the sensitivity to pressure expressed in nm/MPa, while γ is the contrast of the intermodal interference fringes. As the sensor output of the two polarizations sum-up, the overall intensity can be expressed as:

(13) $$I\left(\lambda ,p\right)=I_{0}{\left(\lambda -\lambda_{0}^{av}-K_{p}^{av}p\right)}^{2}\gamma +\text{const}$$

where $\lambda_{0}^{av}$ and $K_{p}^{av}$ indicate the position of the interference minimum at zero pressure and the sensitivity to pressure for interference summative signal, respectively, and are expressed by the following relations:

(14) $$\lambda_{0}^{av}=\lambda_{0}^{x}\cos^{2}\alpha +\lambda^{y}\sin^{2}\alpha$$

(15) $$K_{p}^{av}=K_{p}^{x}\cos^{2}\alpha +K_{p}^{y}\sin^{2}\alpha$$

Equation (14) explains the dependence of the position of the interference minimum at zero pressure upon the azimuth of the input polarization (Figure 8d). Moreover, according to Equation (15), the sensitivity to pressure of the intermodal interference fringes summed-up versus polarizations can be significantly modified by changing the azimuth of the input polarization state, which is in qualitative agreement with the experimental observations presented in Figure 8b. In particular, as per Equation (15), the azimuth of the input polarization state α = 48° yields zero sensitivity to pressure, which is in qualitative agreement with the experimental observations. In the experiment, the zero sensitivity to pressure was obtained for α = 34°. The discrepancies between the experimental result and the prediction based on Equation (15) were most probably related to different contrasts of intermodal interference fringes for *x*- and *y*-polarization caused by the unequal excitation of polarization modes at laterally shifted splices.

**Figure 9:**
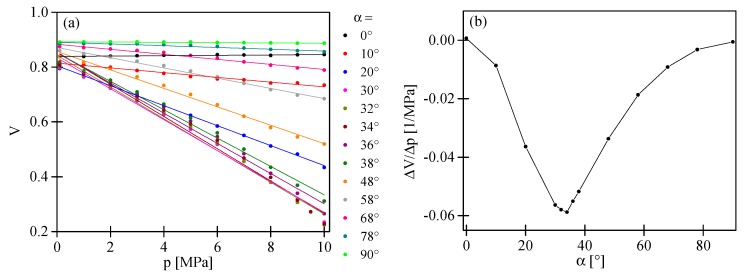
Variation of the intermodal fringe visibility versus applied pressure (**a**) and corresponding sensitivity (**b**) for different azimuths of the input polarization state.

Different values of the intermodal fringes and the envelope sensitivity caused a variation of the interference fringes visibility versus applied pressure. If we selected a high contrast fringe (in our case the fringe with an intensity minimum close to 1563 nm; Figure 7d), the dependence of the visibility versus applied pressure in the range of 0.1–10 MPa was linear and strongly depended on the azimuth of the input polarization state (Figure 9b). The greatest visibility variation was observed for the azimuth of the input polarization state α = 34°. Additionally, as an example, we present in Figure 7e,f the interferometer response for the azimuth of the input polarization state set at α = 58°. For such an excitation, the intermodal fringes moved towards shorter wavelengths, while the envelope towards longer wavelengths was in response to increasing pressure. The displacement of the envelope as well as the intermodal fringe was linear in the investigated range of pressure changes with no trace of hysteresis. The sensitivity coefficients were significantly different and equal to 2.14 nm/MPa and −0.137 nm/MPa for the envelope and the intermodal fringe of the highest contrast, respectively. For α = 58°, we also observed a linear dependence of the intermodal fringe visibility versus the applied pressure with a sensitivity coefficient equal to ∆V/∆p = −0.019 MPa^−1^.

**Figure 10:**
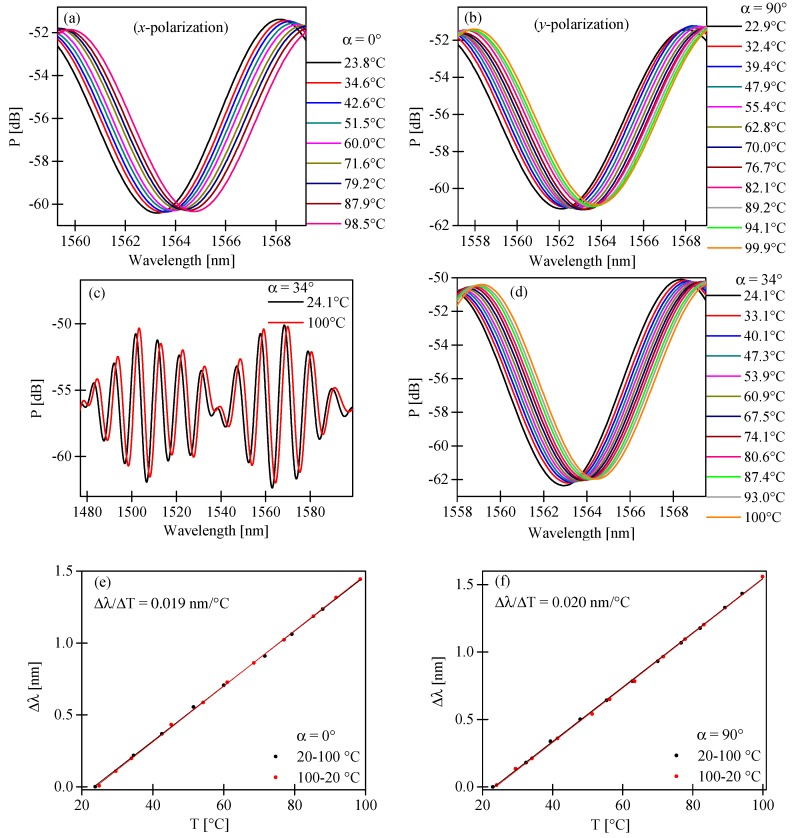
Displacement of the intermodal interference fringe with the intensity minimum close to 1563 nm for increasing temperature in the range of 20–100 °C and azimuth of the input polarization state set at α = 0° (**a**); α = 90° (**b**); and α = 34° (**d**). Output spectrograms registered for the lowest and the highest values of temperature showing the displacement of the envelope for α = 34° (**c**). Displacement of the intermodal interference fringe with an intensity minimum close to 1563 nm for increasing and decreasing temperature for the azimuth of the input polarization state set at α = 0° (**e**); and α = 90° (**f**).

Similar experiments were performed for changes in temperature. As shown in Figure 10a,b,d, for increasing temperature, the intermodal interference fringes moved towards longer wavelengths for *x*- and *y*-polarized modes. A similar response to temperature was observed for the input polarization state set at α = 30°, for which the intermodal sensitivity to pressure was close to null. Moreover, the fringes envelope, determined by the difference in birefringence of the fundamental and the first order mode, moved towards longer wavelengths for increasing temperature (Figure 10c).

Similarly, for hydrostatic pressure, the displacement of the intermodal fringes was linear in the investigated range of temperature with no trace of hysteresis. The temperature sensitivity coefficients for *x*- and *y*-polarized modes had the same sign and almost the same values, $K_{T}^{x}$ = 0.019 nm/°C and $K_{T}^{y}$ = 0.020 nm/°C, Figure 10e,f, respectively. For the azimuth of the input polarization state set at α = 30°, the intermodal fringes moved towards longer wavelengths with a sensitivity coefficient equal to $K_{T}^{av}$ = 0.0195 nm/°C. Similarly, the envelope moved towards longer wavelengths with a sensitivity of 0.02 nm/°C. In the case of temperature, the intermodal sensitivity was almost independent of the azimuth of the input polarization state. Moreover, changes in the fringe visibility caused by the temperature increase in a range from 20–100 °C were practically undetectable. 

The performed measurements showed that for hydrostatic pressure, the sensitivity coefficients for the intermodal fringes and their visibility strongly depended on the azimuth of the input polarization state. This was in contrast to temperature sensitivity, which was practically independent of the azimuth of the input polarization state. The measured values of the sensitivities to temperature and pressure for different azimuths of the input polarization state are shown in Table 1.

The experimental data in Table 1 clearly show that for an appropriate azimuth of the input polarization state, for example for α = 58°, one could obtain significantly different sensitivity values of the intermodal fringes and the envelope in the case of hydrostatic pressure, and the same values in the case of temperature. As the sensitivity coefficients for pressure and temperature were significantly different, the proposed sensor provided the possibility of the simultaneous measurement of these parameters by interrogating two out of three possible quantities, i.e., the displacement and visibility of the intermodal fringes and the displacement of their envelope. As explained in Reference [20], two-parameter sensing could be accomplished by solving a set of linear equations describing the changes in the interrogated quantities observed in response to a variation in the measured physical parameters. To obtain good accuracy in the two-parameter measurements, the sensitivity coefficient 2 × 2 matrix must be well-conditioned. The data presented in Table 1 show that this requirement was fulfilled by our sensor in both the temperature and the hydrostatic pressure measurements.

## 4. Conclusions

In this work, we studied the sensitivity to hydrostatic pressure and to temperature of the in-line Mach-Zehnder interferometer fabricated in a two-mode highly birefringent boron-doped microstructured fiber. The operations principle of the proposed sensor exploits the effects of intermodal interference arising between the fundamental LP_01_ and the first order LP_11_ mode. Due to high fiber birefringence, the intermodal interference fringes were modulated in visibility for differences in the azimuth of the input polarization state from 0° and 90°. The proposed interferometer was tested for measurements of hydrostatic pressure and temperature for different alignments of the input polarizer. Due to asymmetric fiber geometry, the sensitivities to hydrostatic pressure of the intermodal interference fringes for *x*- and *y*-polarizations had an opposite sign and were equal to 0.229 nm/MPa and −0.179 nm/MPa, respectively. We further demonstrated that in the case of hydrostatic pressure, the sensitivity of the intermodal fringes and their visibility was strongly dependent on the azimuth of the input polarization state, while the sensitivity of the envelope remained constant. For the azimuth of the input polarization state equal to α = 34°, the sensitivity of the envelope equaled 2.14 nm/MPa, the sensitivity of the intermodal fringes was equal to zero, while the visibility of the fringes varied in response to pressure changes at a rate of −0.059 MPa^−1^. For α = 58°, the sensitivities of the intermodal fringes and their visibility were significantly different and equaled −0.137 nm/MPa and −0.019 MPa^−1^, respectively. 

For temperature, the intermodal sensitivities for *x*- and *y*-polarized modes had almost the same values, and were equal to 0.019 nm/°C and 0.020 nm/°C for *x*- and *y*-polarization, respectively. Consequently, the average sensitivity $K_{T}^{av}$ was weakly dependent on the azimuth of the input polarization state. Moreover, temperature-induced changes in the fringes visibility were negligible for any alignment of the input polarization state.

As in the case of hydrostatic pressure, the sensitivity of the intermodal fringes and their visibility were strongly dependent on the azimuth of the input polarization α and was practically independent of α in the case of temperature. Furthermore, one could adjust the sensor response to allow for simultaneous measurements of temperature and pressure by interrogating two out of three possible parameters, i.e., the displacement and the visibility of intermodal fringes and the displacement of their envelope.

## Figures and Tables

**Table 1:** Measured values of the sensitivities to temperature and pressure for different azimuths of the input polarization state.

α	Hydrostatic Pressure, Range 0.1–10 MPa			Temperature, Range 20–100 °C		
	Intermodal Sensitivity (nm/Mpa)	Envelope Sensitivity (nm/Mpa)	Fringes Visibility Change (1/Mpa)	Intermodal Sensitivity (nm/°C)	Envelope Sensitivity (nm/°C)	Fringes Visibility Change (1/°C)
0°	0.229	---	0	0.019	---	0
90°	–0.179	---	0	0.020	---	0
34°	0	2.14	−0.059	0.0195	0.02	0
58°	–0.137	2.14	−0.019	0.0195	0.02	0
